# Special Issue: Non-Destructive Testing of Materials and Parts—Techniques, Case Studies and Practical Applications

**DOI:** 10.3390/ma18143312

**Published:** 2025-07-14

**Authors:** Luis M. P. Durão, Nuno C. Loureiro

**Affiliations:** 1 ISEP, Polytechnic of Porto, rua Dr. António Bernardino de Almeida, 4249-015 Porto, Portugal; 2 INEGI Inst. de Ciência e Inovação em Eng. Mecânica e Eng. Industrial, R. Dr. Roberto Frias 400, 4200-465 Porto, Portugal; n.loureiro@doc.isvouga.pt; 3 ISVOUGA, Instituto Superior de Entre Douro e Vouga, R. António de Castro Corte Real, Apart. 132, 4520-181 Santa Maria da Feira, Portugal

The simplest definition of Non-Destructive Testing (NDT) is to “Inspect or measure without doing harm”. From simple visual inspection to the use of lasers, NDT methods cover a broad range of uses in diverse environments. For example, in daily life, we practice visual inspection when we check the thread of a screw; our luggage is inspected by radiography when we go to an airport; and many medical diagnoses rely on echography, an ultrasound method. Thus, NDT provides security, reliability and sustainability in our technological society.

In fact, due to its ability to evaluate the properties of a material, component or system without causing damage, NDT ensures safety in industries like aerospace, nuclear, oil and gas, and construction, where structural integrity is vital; preserves assets by allowing for regular inspections of infrastructures; and supports quality control. Furthermore, NDT is cost-effective, as components can be tested without being destroyed, avoiding costly repairs, replacements or catastrophic failures, and contributing to sustainability by minimizing waste and conserving resources. Despite its key role in many industries, NDT faces several challenges that can impact the accuracy, efficiency and overall effectiveness of inspections.

Current challenges faced by NDT include the detection of tiny or subsurface flaws, especially in complex geometries or hard-to-reach areas; the problems posed by high material complexity, like composites or highly attenuative/anisotropic materials; low Signal-to-Noise ratios, which make it hard to differentiate actual defect signals from background noise; and subjectivity issues, as the interpretation of NDT results still relies on technicians’ experience.

This Special Issue, titled Non-Destructive Testing of Materials and Parts: Techniques, Case Studies and Practical Applications, aims to serve as a forum for presenting the latest research and developments in this field, present new ideas that could help to close knowledge gaps, and highlight future research directions to foster future developments on this interesting topic.

As Guest Editors of this Special Issue, following a rigorous peer-review process of manuscripts received over 16 months, we are pleased to announce the publication of 10 manuscripts. The accomplishment of this Special Issue was only possible due to the interest from proficient researchers from all over the world. The Guest Editors would like to thank all of the authors of the published works and also express their gratitude to the reviewers for their time, and for their valuable comments and suggestions, which improved the quality and value of this Special Issue. The success of this Special Issue would not have been possible without the support of the Section Managing Editor, Ms. Serena Shi, who we thank for her dedication and commitment. The Guest Editors also thank the Editors-in-Chief of *Materials* for this opportunity for collaboration, and congratulate them on their stewardship of such a globally respected journal. The diligence, creativity, and dynamic cooperation of all those mentioned above contributed to the success of this Special Issue.

Several recently published papers focus on the contributions and applications of current Non-Destructive Testing techniques, including Machine Learning, which can be easily combined with other NDT techniques, like acoustic emission (AE) [1] or AE plus radiography, for further data analysis [2]. The monitoring of structures remains an essential issue in NDT, independently of technical fields, from oil and gas [3] to aerospace [4]. Complex materials, like composites, are a recurring theme in NDT studies [2,5], and are also the subject of several thorough reviews of sensing technologies [6], showing the importance of these techniques in detailed defect detection.

We invite the reader to peruse the entire book to become acquainted with the themes of focus in the 10 published papers. One study discusses the use of modern methods, such as acoustic emission combined with machine learning, to conduct effective structural health monitoring, while another presents a method that provides a more effective approach to early fatigue-damage detection by capturing nonlinear Lamb waves, and another investigates the use of laser ultrasonic wave techniques as innovative visualization methods for damage detection. In addition, one study investigates how high-resolution ultrasound can be used to quantify sub-surface wrinkles, for instance, in a CFRP laminate, while another discusses the possibilities presented by combining NDT methods for damage detection in FRP-reinforced elements.

However, there is also some room for studies on the development of electromagnetic tests to study mechanical and thermal features in ferrous alloys, or advances in the use of nanoindentation and sclerometry to evaluate the effects of machining processes on the mechanical characteristics of metallic parts.

Finally, the use of NDT in carbon fiber-reinforced polymers should not be disregarded, as these materials are becoming more important in terms of their applications; both thermal methods and enhanced radiography or image processing have been used for studying machining outcomes like damage extension or other features related to material removal processes primarily developed for metal machining. These recent improvements could contribute to improving the reliability of structural parts made from carbon fiber-reinforced polymers.

As Guest Editors, we hope this Special Issue provides readers with some new insights on the use of Non-Destructive methods like acoustic emission, ultrasound, electromagnetic waves, thermal imaging, nanoindentation, thermography and enhanced radiography.
